# About international federation of societies of endoscopic surgeons

**DOI:** 10.4103/0972-9941.25669

**Published:** 2006-03

**Authors:** Tehemton E Udwadia

**Affiliations:** Dept. of Minimal Access Surgery, P. D. Hinduja National Hospital, Room 2103, Veer Savarkar Marg, Mahim, Mumbai - 400016, India

IFSES is a Federation of Societies representing laparoscopic surgeons in Continents or Countries with large M.A.S. input. Acceptance of Societies is on application which is carefully scrutinised and voted on by ballot at Executive Committee Meetings – the latest Society to be accepted in this International Federation (and creditably on the first ballot) is I.A.G.E.S. Members of the various Societies which form the Federation have representation in and dialogue with IFSES through two nominated Representative Members of each Society, who represents that Society in the IFSES Executive Committee.

The Member Societies are: 1) European Association for Endoscopic Surgery (EAES), 2) Canadian Association of General Surgeons (CAGS), 3) Federation Latinoamericana de Cirugia (FELAC), 4) Association Latinoamaericana de Cirujanos Endoscopistas (ALACE), 5) Japan Society for Endoscopic Surgery (JSES), 6) Society of American Gastrointestinal Endoscopic Surgeons (SAGES), 7) Endoscopic and Laparoscopic Surgeons of Asia (ELSA), 8) Indian Association of Gastrointestinal Endo-Surgeons (IAGES), 9) Chinese Society for Laparo-Endoscopic Surgery (CSLES), and 10) General Surgeons of Australia (GSA).

The only continent not yet part of IFSES is Africa and it is expected that a Pan African Society of M.A.S. will take its place in IFSES.

A unique concept of IFSES was that these surgeons from several Endoscopy Societies sought and obtained the partnership of Industry, so that leading manufacturers joined IFSES as Corporate Members and are part of the Executive Committee making this the only Federation where surgeons integrate with and actively involve Industry to help achieve the mutual goal of the growth and spread of M.A.S. The current Corporate Partners are: 1. Aesculap AG. C.O KG, 2. Ethicon Endo-Surgery, 3. Karl Storz GmbH and Co, 4. Olympus, 5. Tyco-US Surgical

M.A.S. is so encompassing in its spread, so varying in its complexity and instrumentation, the spectrum of surgical care in the world so disparate, from the opulent to the deprived, that to state that the IFSES mission through the activities of its Member Societies and Corporate Members is the growth and spread of M.A.S. all over the world may appear idealistic and futuristic. IFSES is aware of the magnitude of the problems, but is evolving a strategy to try, as best, to achieve its mission to ensure that the cutting edge of surgical progress is made available to all people in all places

IFSES is rich in the quality, diversity, capability, activity and culture of each one of its Member Societies and the ethical and professional excellence of its Corporate Members and acts as a meeting point, unifying force, melting pot, apex body of all its Member Societies, for harmonious interaction and cooperative efforts of the Societies, to help facilitate the spread of M.A.S. world wide. The current President of IFSES comes from a developing country and it has been his constant thrust to channelise the efforts of all Member Societies and Corporate Partners towards helping ensure optimal surgical care through M.A.S. to the developing world. With this view a Training Centre is being set-up in Accra, Ghana with the support of Industry. Other Training Centres in the developing world are under evaluation.

One important function is the Biennial World Congress of Endoscopic Surgery. IFSES designates the venue of the World Congress after debating and voting on bids from Member Societies. The next World Congress will be in September 2006 in Berlin under the Chairmanship of Professor Gerhard Buess. The commitment of IFSES towards the developing world is evident from the fact that a full day session on Low-Cost M.A.S. will be he held at this Congress with participation from Governments, the World Health Organisation, Health Care administrators and Surgeons.

A few aspects which IFSES can contribute to spread of M.A.S. worldwide are:

- Record current details of all Member Societies and Corporate Members, their Presidents / Chairmen, contact persons with addresses, representatives in the IFSES Executive Committee, dates of future conferences, publications, websites etc.
- Maintaining and posting a complete calendar of dates of all surgical endoscopic meetings world wide over the next few years, not only of Member Societies but also of National Societies of its Europe [EAES], Asia [ELSA], India [IAGES], Latin America [FELAC and ALACE] Member Societies. This would enable National and International Societies to plan meeting without clash or conflict and give surgeons prior notice of these meetings. Moreover, it would provide Industry an opportunity to exhibit their products with proper planning. Greater participation of industry would give financial support to the Conferences. For this it is very imperative that EVERY Member Society exert to post this information on the Website early and with complete details of conferences venue, dates, host organization, contact persons, registration and abstract dates. Under the leadership and initiative of Ms. Lynn Farrar of Ethicon Endosurgery a comprehensive new website is under preparation.
- Creating an International Data Base of:
- Surgeons and institutions desiring to receive the benefit of M.A.S. training programmes.
- Surgeons of good standing willing to travel and teach in neighbouring areas.
- Surgeons willing to absorb some of these trainees into their Fellowship/ Observer programmes.
- Stockpile of equipment ready for donation when required by Corporate Partners/Member Societies/Hospitals.
- International Fellowship opportunities list – Universities, Hospitals could give date of Fellowship in M.A.S. open to all countries, particularly in the developing World.
- International Reporting of Research Grants in terms of Fellowship Opportunity.
- International job registering in terms of the above. This could include Bio-Engineering, nursing, O.R. technician opportunities in M.A.S.

To maintain and update this information it is mandatory that each Member Society and Corporate Partner transmit all relevant data to the IFSES Secretary to keep the IFSES website IFSES.org informative and vibrant. A conspicuous absentee from the Member Societies is representation from Africa. A Committee has been set-up with Professor J. Perissat who has several years of commitment and experience in developing M.A.S. in that Continent as Chairman to correct this inequity and as also embrace and support countries in Africa which have their own active Endoscopy Surgical Societies.

All the above and much more has for the International Federation of Societies of Endoscopic Surgeons basically only one mission – to ensure safe M.A.S. by well trained surgeons, at affordable cost, world-wide.

